# Coffee and tea consumption and glioma risk: a meta-analysis of cohort studies

**DOI:** 10.3389/fnut.2024.1506847

**Published:** 2024-12-16

**Authors:** Jinyu Pan, Chuan Shao, Hui Tang, Nan Wu

**Affiliations:** ^1^Department of Neurosurgery, Chongqing General Hospital, Chongqing University, Chongqing, China; ^2^Department of Neurosurgery, Nanchong Central Hospital, The Second Clinical Medical College, North Sichuan Medical College, Nanchong, China

**Keywords:** glioma, coffee, tea, cohort, risk factors, meta-analysis

## Abstract

**Background:**

Research on the association between glioma risk and coffee and tea consumption remains inconclusive. This study seeks to present a meta-analysis of the relationship between coffee and tea intake and glioma risk.

**Method:**

Relevant cohort studies that collected coffee and tea exposure prospectively were identified through searches of the PubMed, Embase, and Scopus databases. Eligible studies included those providing adjusted relative risk estimates or hazard ratios (HRs) with 95% confidence intervals (CIs), or data sufficient for such calculations. Study quality was evaluated using the Newcastle-Ottawa Scale, while the GRADE system assessed the quality of evidence. The analysis explored glioma risk concerning the highest versus lowest levels of coffee and tea intake, supplemented by a dose–response evaluation using a one-stage robust error meta-regression model.

**Results:**

A total of nine studies, published between 2004 and 2020, were included. In a model comparing the highest and lowest levels of coffee and tea consumption, 3,896 glioma cases were identified among 2,648,468 participants. Correspondingly, the pooled HRs with 95% CIs were 0.98 (0.87–1.09) for coffee and 0.95 (0.86–1.06) for tea, respectively. Furthermore, no evidence of publication bias was detected for either beverage. The dose–response analysis indicated a near “L”-shaped relationship between tea consumption and glioma risk, with the most notable risk reduction observed in individuals consuming more than 2.5 cups of tea per day. However, additional tea intake beyond this threshold did not confer evident risk reduction. According to Grade scoring system, the quality of meta-evidence was classified as “very low” for coffee and “low” for tea.

**Conclusion:**

This meta-analysis provides evidence suggesting a potential inverse association between tea consumption and glioma risk, while no such association was observed for coffee consumption. Given that the evidence for coffee was classified as “very low” and for tea as “low,” cautious interpretation of the findings is warranted, and further research is needed to validate these results.

## Introduction

Gliomas represent a heterogeneous group of central nervous system (CNS) tumors, characterized by varying degrees of invasiveness and prognosis. According to the 2021 WHO classification of CNS tumors, which organizes gliomas based on histopathological features and distinct molecular biomarkers, these tumors are classified into five primary clusters: pediatric-type diffuse low-grade gliomas, pediatric-type diffuse high-grade gliomas, adult-type diffuse gliomas, circumscribed astrocytic gliomas, and ependymal tumors (1, 2). Gliomas account for 80 to 85% of malignant CNS tumors in adults, making them the most common primary CNS tumors in this population, with an age-adjusted incidence rate of 5.6 per 100,000 person-years in the United States (3, 4).

As with other tumors, research into the etiology and susceptibility factors of gliomas is ongoing, with limited understanding thus far. Advanced age is currently recognized as a significant risk factor (4). In addition, glioma incidence exhibits notable variability across sex, race or ethnicity (1, 5). The highest incidence is observed among Non-Hispanic White people, with a male predominance (1, 5). Beyond exposure to ionizing radiation, which remains the only well-established environmental risk factor, a history of allergies or atopic disease has been consistently associated with a reduced risk of glioma, albeit with limited supporting evidence (6).

Coffee and tea are enjoyed across diverse cultures and regions with an estimated consumption of 2.25 billion cups of coffee (7) and approximately 3 billion cups of tea each day (8). Both drinks are rich in dietary sources of bioactive compounds, such as caffeine, polyphenol compounds, amino acids, minerals, and vitamins (9, 10). These compounds are known for their antioxidant properties, anti-cancer potential, and neuroprotective benefits *in vitro* and experimental studies, as summarized in several reviews (9–12). Given the extensive global consumption of coffee and tea, as well as their range of biological effects, there is growing interest in exploring the link between these beverages and health outcomes (13, 14). Currently, epidemiological evidence for the association between glioma risk and coffee and tea consumption is inconsistent (15–30). This discrepancy may be associated with different study methodologies, such as sample size, recall bias, and residual confounders. Several meta-analyses or systematic reviews have tried to settle this issue (31–37). Notably, previous meta-analyses included some retrospective case–control studies (31, 34, 36, 37), unadjusted risk estimates (33, 34, 36, 37), multiple reports from the same cohort (37), and missed earlier prospective studies in their analysis (35–37). To overcome these limitations, we performed an updated meta-analysis of cohort studies which collected coffee and tea exposure prospectively to quantitatively summarize the relationship.

## Method

### Report guideline

This meta-analysis was designed, conducted, and documented in accordance with the Preferred Reporting Items for Systematic Reviews and Meta-Analyses statement (38). There was no registered protocol.

### Literature search

We carried out a comprehensive search across the PubMed, Embase, and Scopus databases, covering the period from their inception to November 17, 2024. The following keywords were utilized: coffee, tea, diet, beverages, drinking, glioma, brain tumors, brain cancer, brain neoplasms, cerebral cancer, cerebral tumors, cerebral neoplasms, intracranial cancer, central nervous system cancer, intracranial neoplasms, intracranial tumors, central nervous system tumors, and central nervous system neoplasms. Detailed search strategies are outlined in Supplementary Table 1. We also conducted a meticulous manual review of the reference lists of pertinent articles, reviews, and meta-analyses to identify any studies that may have been missed by the initial search terms.

### Study selection

The criteria for inclusion were defined as follows: (1) cohort studies that examined coffee or tea consumption as the primary exposure variable and glioma as the outcome, and (2) studies that reported adjusted relative risk estimates or hazard ratios (HRs) along with 95% confidence intervals (CIs) or provided data necessary for such calculations. Exclusion criteria comprised: (1) letters and conference abstracts lacking original data, (2) studies that addressed total brain tumors, (3) retrospective case–control studies, and (4) studies presenting only crude risk estimates with 95% CIs. In our study, we also included those with a retrospective cohort design in a population cohort which collected coffee and tea exposure prospectively. The study selection was performed by JP and checked by CS.

### Data extraction

Two investigators (JP and CS) independently retrieved the following information: first author’s last name, year of publication, country in which the research was conducted, name of the cohort, enrollment period, number of cases, total population size, age range of participants, time of follow-up, exposure categories, and the associated risk estimates with 95% confidence intervals (CIs), adjusted for the maximum number of potential confounding variables. Any discrepancies were resolved by discussion.

### Statistical analysis

All statistical analyses were executed utilizing Stata software version 14.0 (StataCorp, College Station, Texas, USA). To evaluate the association between coffee and tea consumption and glioma risk, both categorical meta-analyses comparing the highest and lowest intake levels and dose–response analyses were performed. Pooled effects were calculated utilizing random-effects model when significant heterogeneity was found; otherwise, fixed-effects model was used. The I^2^ statistic was employed to evaluate heterogeneity and an I^2^ value greater than 50% is considered to indicate substantial heterogeneity (39). The possibility of publication bias was evaluated by visually inspecting funnel plots and utilizing the Egger’s test (40). Additionally, sensitivity analyses were performed by systematically omitting individual studies to determine the robustness of the results.

A one-stage robust error meta-regression model (REMR), as outlined by Xu and Doi, was employed to explore the dose–response relationship (41). For the analysis, all consumption measures were normalized to cups per day, with a single cup equating to four ounces of coffee. The REMR approach required at least two exposure levels, each accompanied by their respective relative risks and 95% CIs. In cases where coffee or tea intake was provided as a range, the median or average was utilized to estimate the exposure level. If these values were not available, the midpoint of the range was utilized instead. For the highest open-ended category, the width was assumed to match the interval of the preceding category, while the lower boundary for the lowest open-ended category was set to zero.

Kuan et al. conducted a pooled analysis that encompassed three larger cohorts: the UK Million Women Study, the National Institutes of Health–AARP Diet and Health study (NIH-AARP), and the Prostate, Lung, Colorectal, and Ovarian (PLCO) Cancer Screening Trial (28). However, this study did not provide detailed exposure levels for coffee and tea (28). In contrast, the studies by Dubrow et al. (24) and Hashibe et al. (26), which utilized data from the NIH-AARP Study and the PLCO study, respectively, offered comprehensive exposure level data. To optimize data utilization and prevent redundant data usage, we incorporated Kuan et al.’s research (28) into the comparative analysis between the highest and lowest exposure groups. Additionally, the studies by Dubrow et al. (24) and Hashibe et al. (26) were included in the dose–response evaluation (24, 26).

### Study quality

For study quality assessment, our study utilized the Newcastle-Ottawa Scale (NOS: available at https://www.ohri.ca/programs/clinical_epidemiology/oxford.asp, accessed on November 18, 2024), which evaluates studies based on three domains: selection (four items, one star per item), comparability (one item, up to two stars), and outcome (three items, one star per item). In our study, we made the following definitions: Considering that confounding factors are one of the biggest concerns in observational studies, for the “Comparability” item, a study can be awarded a maximum of one star under this sub-item if it adjusts for age or birth age in the analysis. A study with follow-up (greater than the median or mean follow-up of 5 years, or with a maximum follow-up period of 10 years or more) was awarded one star. If a study collected exposure data using questionnaires with validated reliability, it was awarded one star. Since universally accepted formal criteria for high quality have not yet been defined, the thresholds for converting the Newcastle-Ottawa scales to Agency for Healthcare Research and Quality (AHRQ) standards classify studies as good quality if they score 3–4 stars in the selection domain, 1–2 stars in the comparability domain, and 2–3 stars in the outcome domain; fair quality if they score 2 stars in the selection domain, 1–2 stars in the comparability domain, and 2–3 stars in the outcome domain; and poor quality if they score 0–1 star in the selection domain, 0 stars in the comparability domain, or 0–1 star in the outcome domain.

### Grading quality of evidence

The Grade scoring system was utilized to evaluate the quality of meta-evidence in this study (42). Evidence from cohort studies is initially classified as “Low.” The quality of evidence may be upgraded based on factors such as a substantial magnitude of effect, the presence of plausible residual confounding unlikely to diminish the effect size, and the existence of a dose–response gradient. Conversely, it may be downgraded due to inconsistency, indirectness, imprecision, or publication bias. Ultimately, the evidence quality is categorized into four levels: high, moderate, low, and very low.

## Results

### Basic characteristic

**Figure 1:**
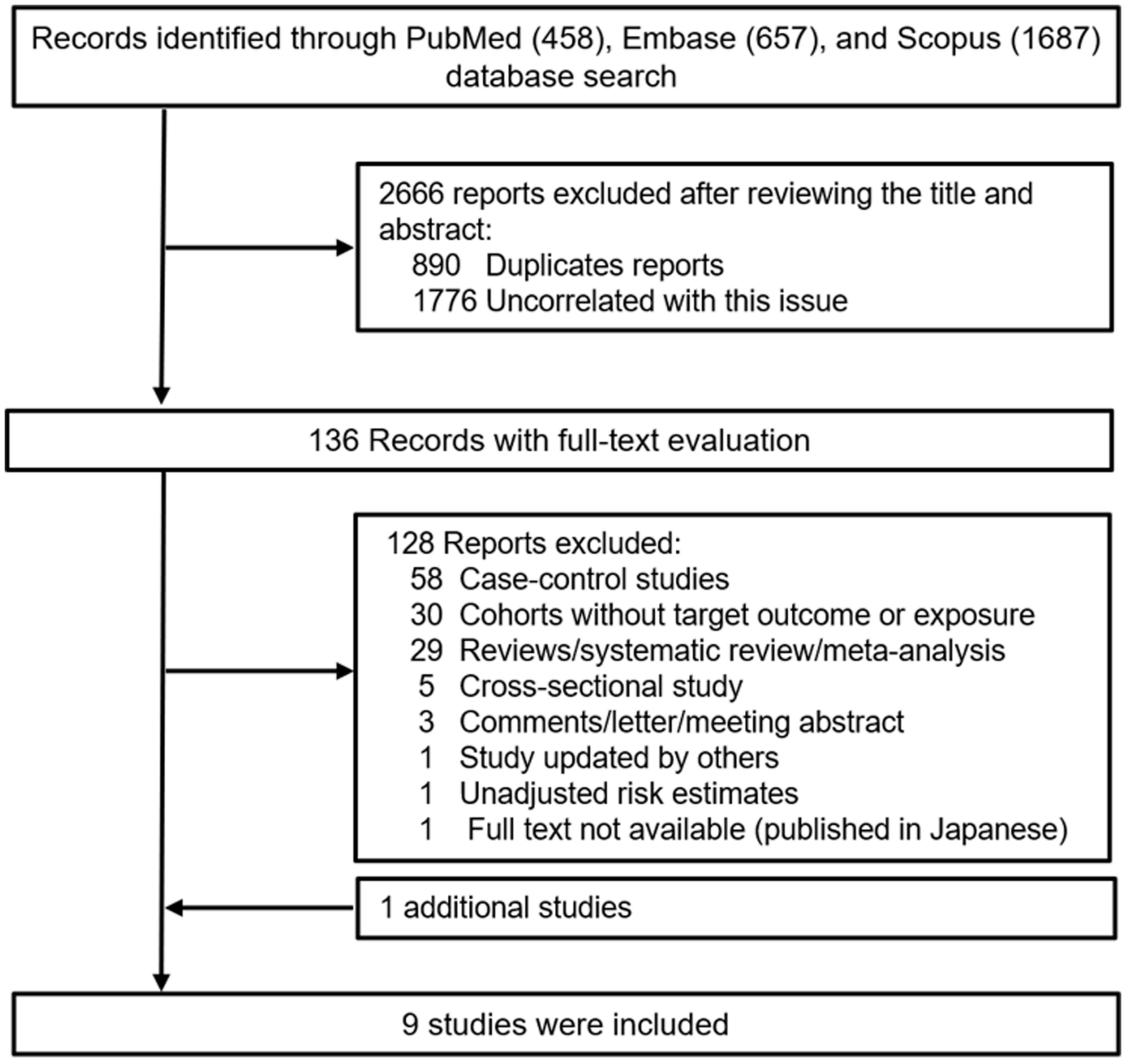
Flowchart of identifying studies.

Figure 1 outlines the process for the identification and selection of literature. The initial database search yielded 458 records from PubMed, 657 records from Embase, and 1,687 records from Scopus. Following a title and/or abstract screening, 890 duplicates and 1776 records uncorrelated this issue were excluded. Upon full-text review, 128 of the remaining 136 reports were excluded, leaving eight studies that met the inclusion and exclusion criteria. Additionally, one was located through manual reference list reviews (25). Ultimately, nine studies were incorporated into the meta-analysis (20, 21, 24–30). Table 1 and Supplementary Table 2 presents the primary characteristics of the studies. These studies were published between 2004 and 2020, with the majority conducted in the United States (20, 24–26, 30), alongside one in Japan (27), one in the UK (26), and one across several European countries (21). The age of participants at the time of cohort enrollment was ≥25 years. Case identification relied on cancer registries, national death indices, hospital records, the National Health Service Central Registers, or unspecified medical records, with all cases diagnosed using the International Classification of Diseases ninth or tenth revision (ICD-9/10) and/or the International Classification of Diseases for Oncology (ICD-O) codes. Coffee and tea consumption was assessed through a variety of questionnaires. Several studies have validated dietary data using various methods, including 24-h dietary recalls (21, 25), 7-day dietary records (25, 28), two 24-h recalls (24, 28), and 14- or 28-day dietary records (27), as well as two 1-week diet records (30). In the PLCO study, a valid questionnaire was used, although no detailed methodology was reported (26). In the remaining two studies, the reliability of the questionnaires could not be confirmed from the original reports (20, 29).

**Table 1:** Basic characteristic.

Study	Country	Cohort name	Cases/cohorts	Baseline age	Follow-up time	Glioma identification	Exposure method
Efird et al. (20)	United States	KPMCP-NC	130/133,811	≥ 25	13.2 years (mean)	Cancer registry with ICD-9 and ICD-O codes	Questionnaire
Michaud et al. (21)	European	EPIC	343/521,448	25–70	8.5 years (mean)	Cancer registry with ICD-O-2 codes	Food frequency questionnaire with validity
Dubrow et al. (24) ^*^	United States	NIH-AARP	904/545,771	50–71	10.6 years (median)	Cancer registries with ICD-O-3 codes	Food frequency questionnaire with validity
Nelson et al. (25)	United States	HHP/HAAS	9/8,006	45–68	Approximately 30 years	Medical records with ICD-9 codes	Lifestyle factors questionnaire with validity
Hashibe et al. (26) ^*^	United States	PLCO	103/97,334	55–74	2001–2011	Medical records with ICD-O-2 codes	Diet history questionnaire with validity
Ogawa et al. (27)	Japan	JPHCS	60/106,324	40–69	18.1 years (mean)	Cancer registry or hospitals records with ICD-O-3 codes	Health habits frequency questionnaire with validity
Kuan et al. (28)^#^	The UK	MWS	1,173/692,176	50–64	2000–2015	Cancer registry with ICD-10 and ICD-O-3 codes	Semi-quantitative dietary questionnaire or an online 24-h dietary recall questionnaire with validity
	United States	NIH-AARP	1,005/470,780	50–69	1995–2011	NDI and cancer registry with ICD-10 and ICD-O-2 codes	Food frequency questionnaire with validity
	United States	PLCO	135/99,148	55–74	1998–2009	NDI or medical records with ICD-10 and ICD-O-2 codes	Diet history questionnaire with validity
Creed et al. (29)	The UK	The UK Biobank	487/379,259	40–69	5.8 years (median)	National Health Service Central Registers with ICD-10 codes	Questionnaire
Cote et al. (30)	United States	NHS	256/92,389	30–55	1980–2013	NDI or medical records with ICD-9 CM codes	Food frequency questionnaires with validity
	United States	NHSII	87/95,242	25–42	1991–2013	NDI or medical records with ICD-9 CM codes	Food frequency questionnaires with validity
	United States	HPFS	211/49,885	40–75	1986–2016	NDI or medical records with ICD-9 CM codes	Food frequency questionnaires with validity

KPMCP-NC, Kaiser Permanente Medical Care Program of Northern California; EPIC (Denmark, Italy, the Netherlands, Norway, Spain, Sweden, and the United Kingdom. For France, Germany, and Greece), the European Prospective Investigation into Cancer and Nutrition; NIH-AARP, the NIH-AARP Diet and Health Study; HHP/HAAS, the Honolulu Heart Program/ the Honolulu-Asia Aging Study; PLCO, the Prostate, Lung, Colorectal, and Ovarian cancer screening trial; JPHCS, the Japan Public Health Center-Based Prospective Study; HPFS, Health Professionals Follow-up Study; NHS, Nurses’ Health Study; NHSII, Nurses’ Health Study II; MWS, Million Women Study; NDI, national death index; ICD, International Classification of Diseases; ICD-O, International Classification of Diseases -Oncology; UK, United Kingdom.

* The two studies were only included in dose–response analysis.

# This study was only included in the highest versus lowest analysis.

### Coffee consumption and glioma risk

**Figure 2:**
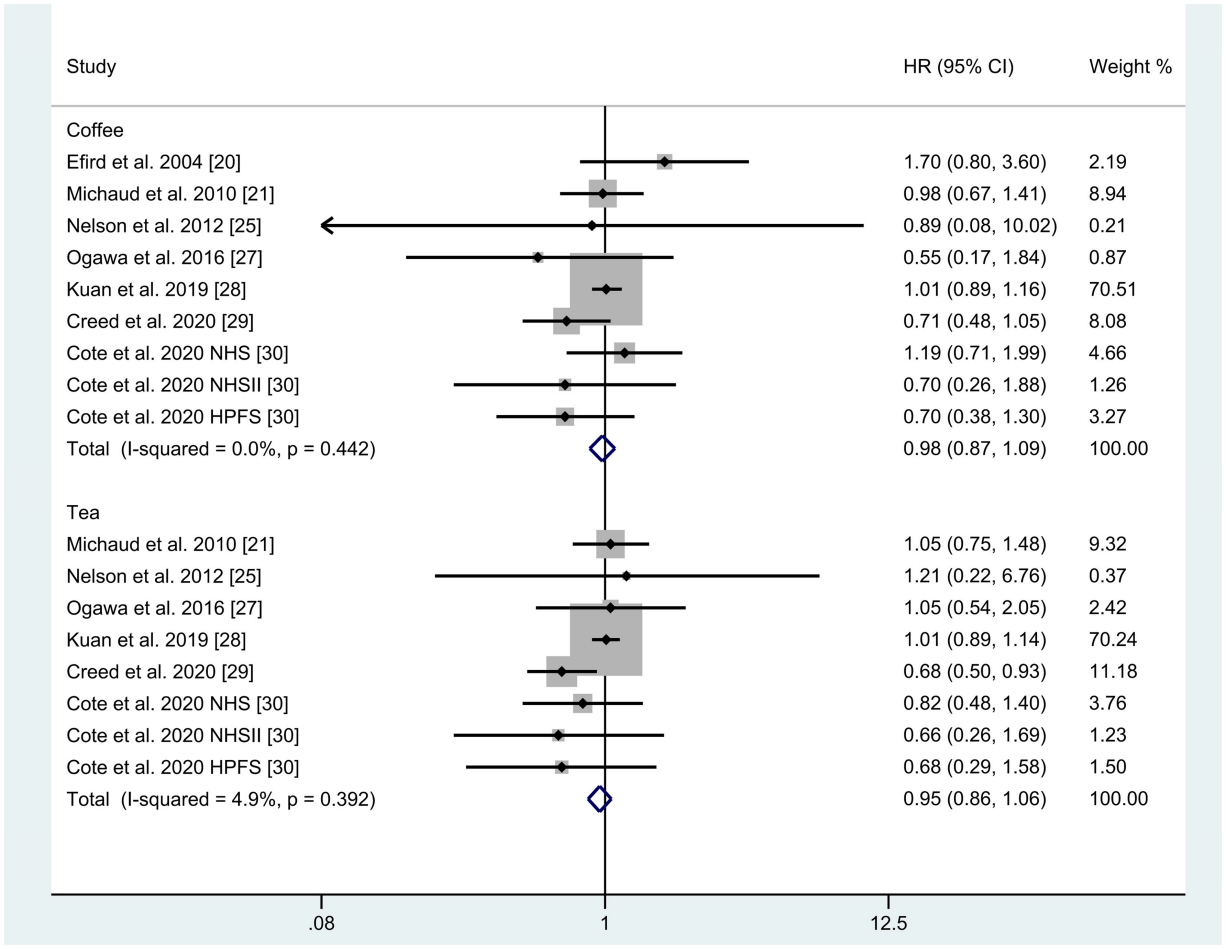
Forest plots for the relationship between coffee and tea intake and glioma risk.

**Figure 3:**
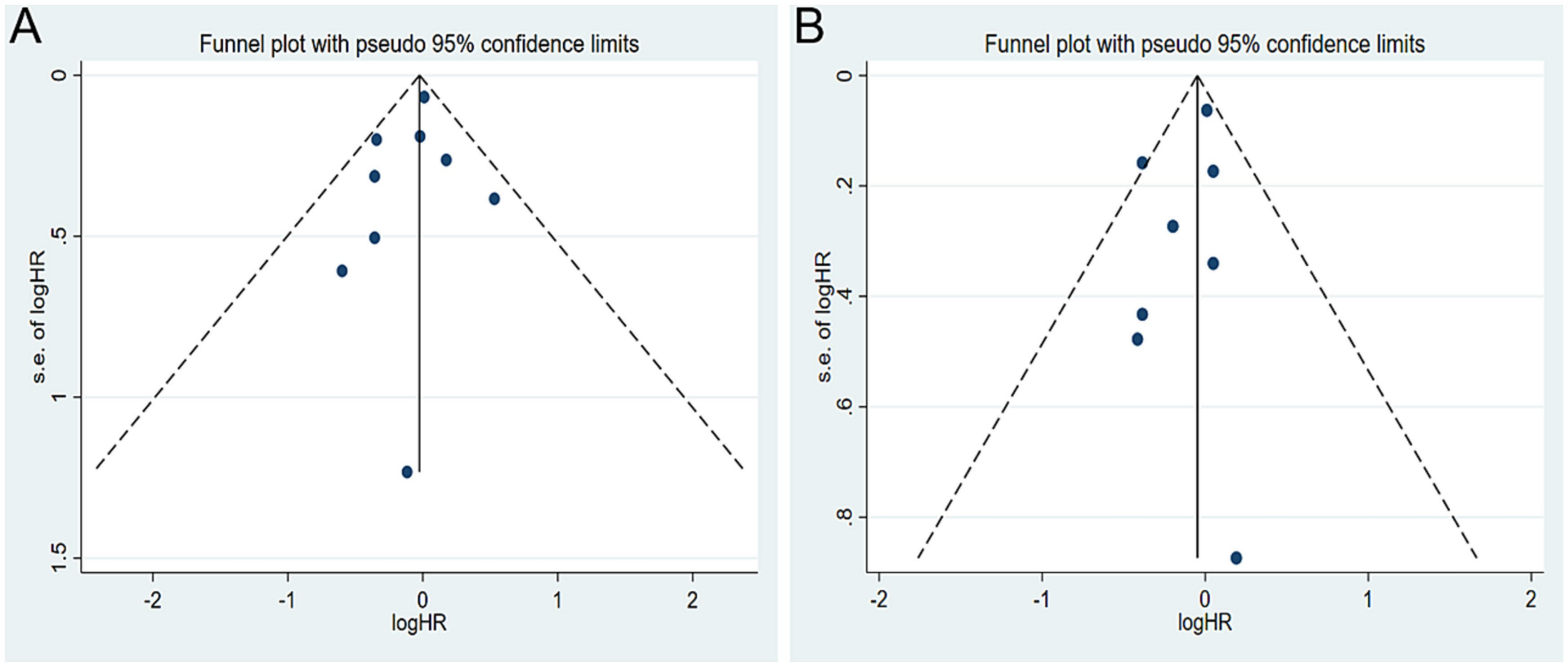
Funnel plot for publication bias. **(A)** Coffee; **(B)** tea.

**Figure 4:**
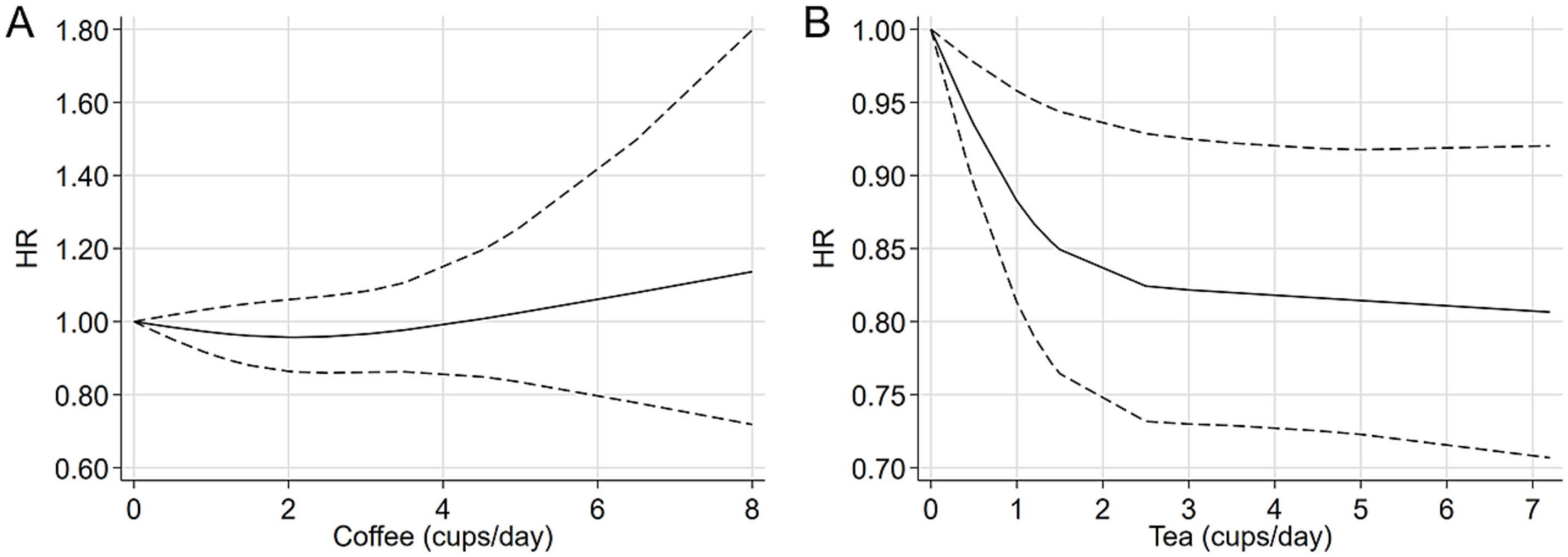
Dose–response relationship. **(A)** Coffee; **(B)** tea.

A total of seven studies were identified for the comparison between the highest and lowest levels of coffee consumption (20, 21, 25, 27–30). Figure 2 illustrates the HRs and 95% CIs for each individual study, along with the overall analysis. Heterogeneity test suggested that no significant heterogeneity was detected across the studies (I^2^ = 0.0%, *p* = 0.442) and fixed-effects model was used. The aggregated HR was found to be 0.98 (95% CI 0.87, 1.09). Sensitivity analyses confirmed that no single study had a considerable impact on the overall results (Supplementary Table 3), with the pooled HR ranging from 0.90 (95% CI 0.73, 1.10) to 1.00 (95% CI 0.89, 1.13). The symmetry of the funnel plots suggests the absence of publication bias (Figure 3A), a finding further supported by formal statistical testing (*p* for Egger’s test = 0.463). In a dose–response analysis, eight studies met the inclusion criteria (20, 24–27, 29, 30). A linear association between coffee intake and glioma risk was identified (Figure 4A, *p* = 0.656). We also perform a sensitivity analysis limited to those studies which reported adjusted risk estimates for smoking. Correspondingly, the pooled HR was 0.98 (95% CI 0.87, 1.09) in model comparing the highest and lowest levels of coffee consumption. The *p* for dose–response analysis was 0.457 (Supplementary Figure S1A).

### Tea consumption and glioma risk

A total of six studies were included in the highest versus lowest comparison of tea consumption (21, 25, 27–30). Figure 2 illustrates the study-specific HRs with their 95% CIs, as well as the overall result. There was no statistically significant heterogeneity detected among the studies (I^2^ = 4.9%, *p* = 0.392) and fixed-effects model was used. The pooled HR was calculated as 0.95 (95% CI 0.86, 1.06). Sensitivity analyses indicated a marginal association between tea consumption and glioma risk (HR 0.83, 95% CI 0.69, 1.01, I^2^ = 0.0%, *p* = 0.605, Supplementary Table 4) when a pooled analysis of three large cohorts was excluded (28). Both the funnel plots (Figure 3B) and Egger’s test (*p* = 0.284) did not indicate publication bias. Six studies contained adequate data for conducting a dose–response analysis between tea consumption and glioma risk (24–27, 29, 30). The analysis demonstrated a nonlinear relationship (Figure 4B, *p* = 0.034), with a pronounced inverse association observed for individuals consuming more than 2.5 cups of tea per day. However, no evident reduction in glioma risk was observed with further increases in tea consumption beyond this level. Similar to our analysis of coffee, we performed an additional sensitivity analysis for tea, focusing exclusively on studies that reported adjusted risk estimates for smoking. The result was still not significant (HR 0.95, 95% CI 0.86, 1.06) in model comparing the highest and lowest levels of tea consumption. Moreover, a nonlinear relationship was found (Supplementary Figure S1B, *p* = 0.002).

### Study quality and grading quality of evidence

According to the NOS for cohort study quality assessment, all included studies were consider to be good quality (Supplementary Table 5). Furthermore, the quality of meta-evidence was classified as “very low” for coffee and “low” for tea (Supplementary Table 6).

## Discussion

Coffee and tea consumption has been examined in relation to various health outcomes, including glioma (13–30). Despite these efforts, the findings have been inconsistent. To address this, we conducted a meta-analysis of cohort studies to consolidate the available evidence and draw more definitive conclusions regarding the impact of coffee and tea consumption on glioma risk. In this comprehensive meta-analysis, no significant associations were observed between coffee or tea consumption and glioma risk when comparing the highest versus lowest intake categories. Additionally, dose–response analysis indicated a nonlinear association between tea consumption and glioma risk.

Several studies have addressed the specific type of coffee (24, 26, 30). In a large-scale, prospective NIH-AARP Diet and Health Study, which encompassed 5,268,995 person-years of follow-up, no statistically significant relationship was found between caffeinated, decaffeinated, or overall coffee intake and glioma risk (24). These results were corroborated by the PLSO trial (26), as well as a pooled analysis from the Health Professionals Follow-up Study (HPFS), the Nurses’ Health Study (NHS), and NHS II (30). Additionally, three separate investigations examined the link between caffeine intake and glioma risk (24, 26, 30). Both the NIH-AARP Study (24) and the PLCO study (26) reported no significant HRs when comparing the highest and lowest quintiles of caffeine consumption, nor were there significant trends showing a reduction in glioma risk with increasing caffeine intake. However, a borderline association was observed in the combined cohorts of NHS, NHS II, and HPFS (30). To the best of the authors’ knowledge, most studies failed to distinguish between different types of tea, such as green and black tea, with the exception of a study conducted in Japan that focused on green tea (27). The varying degrees of fermentation between green and black tea may result in differing health effects (43). Non-fermented green tea is high in catechins and contains minimal theaflavins, whereas fully fermented black tea has low catechin content but is rich in theaflavins (43, 44). Additionally, the method of coffee preparation can influence its chemical composition (45). For instance, the diterpene levels in filtered coffee are minimal in comparison to those found in boiled or French press coffee (45). Therefore, the types of coffee and tea, along with their respective brewing methods, require further investigation.

The link between coffee and tea consumption and a reduced risk of developing glioma is biologically feasible. Both tea and coffee are rich in a variety of biologically active compounds, such as caffeine, polyphenols, and flavonoids (9, 10). *In vitro* research has shown that caffeine may suppress the proliferation of glioma cells via the PKA/GSK3β pathways (46) and inhibit cellular migration through the ROCK-FAK pathway (47). Resveratrol, a non-flavonoid polyphenol compound, has been found to suppress glioma cell growth by modulating oncogenic microRNAs as well as the NF-κB and PI3K/AKT/mTOR pathways (48). In animal models of glioma, resveratrol has demonstrated the ability to slow tumor progression (49). Additionally, flavonoids have been shown to delay glioma growth and to enhance the efficacy of chemotherapeutic drugs in combating glioblastoma in a synergistic manner (50, 51). Collectively, these mechanisms suggest that the consumption of tea and coffee may confer protective effects against glioma.

Various meta-analyses have extensively evaluated this topic (31, 32, 34–37). For coffee consumption, almost all prior meta-analyses observed a non-significant inverse association when comparing the highest to lowest consumption levels (31, 36, 37), except an earlier study (34). Regarding tea consumption, earlier meta-analyses published before 2018 reported a non-significant association with glioma risk (31, 32, 34), while more recent meta-analyses identified a significant inverse association (35–37), including a linear association found in 2022 studies (36, 37). Although our findings partially align with those of prior meta-analyses, there are notable differences between our current study and previous research (Supplementary Table 7). Except for the four cohort studies with extended follow-up time updated by others (22, 24), our investigation exclusively incorporated cohort studies where exposure data were prospectively collected. Moreover, a dose–response analysis was conducted using the REMR method (41). Earlier meta-analyses employed the Greenland and Longnecker approach (52), which required the original studies to report the distribution of cases and person-years (non-cases) for at least three exposure levels. In contrast, using the REMR method does not need the distribution of exposure participates and necessitates a minimum of two exposure categories with corresponding relative risks and 95% CIs, thereby allowing for the inclusion of more studies in the dose–response analysis and enabling a more precise estimation of the dose–response curve. Notably, our analysis revealed a near “L”-shaped association between tea consumption and glioma risk, with the pronounced inverse association observed in individuals consuming 2.5 cups of tea per day. Beyond this level, no evident reduction in glioma risk was detected. Finally, we give a judge of the meta-evidence. Based on the current studies, the quality of meta-evidence was classified as “very low” for coffee and “low” for tea.

Our research is subject to several limitations. Firstly, the possibility of residual confounding factors from the original studies cannot be entirely ruled out. Secondly, the potential for misclassification bias warrants consideration. While all the included studies measured coffee intake based on the number of cups consumed per day, a universally accepted standard for coffee cup size does not exist. Furthermore, the variation in exposure levels, particularly between the highest and lowest reference categories, could potentially weaken the observed associations rather than enhance them, as highlighted by Poole et al. (53). Lastly, as with other meta-analyses, concerns regarding potential publication bias may affect the robustness of our findings, although no direct evidence of such bias was detected.

In conclusion, this meta-analysis suggests a potential association between tea consumption and a reduced risk of glioma, while no significant correlation was found between coffee consumption and glioma incidence. However, it is important to note that the evidence for coffee consumption was classified as “very low,” and for tea consumption, it was classified as “low.” Therefore, these findings should be interpreted with caution. Further studies with more robust evidence are warranted to confirm these associations and provide more definitive conclusions.

## Data Availability

The original contributions presented in the study are included in the article/Supplementary material, further inquiries can be directed to the corresponding authors.
