# Supplementary material for: Coffee and tea consumption and glioma risk: a meta-analysis of cohort studies
Source: Front Nutr. 2024 Dec 16;11:1506847. doi: 10.3389/fnut.2024.1506847 (PMC11684387; doi:10.3389/fnut.2024.1506847)
Supplement: Supplementary file 1 [file Data_Sheet_1.DOCX]

**Supplementary Table 1 Data search.**

|  | **PubMed** |  |  |  |  | **Embase** |  |  |  |  | **Scopus** |  |  |
| --- | --- | --- | --- | --- | --- | --- | --- | --- | --- | --- | --- | --- | --- |
| Search | Query | Items found | Date |  | Search | Query | Items found | Date |  | Search | Query | Items found | Date |
| #1 | Search coffee[Title/Abstract] | 20,477 | 2024.11.17 |  | #1 | Search 'coffee':ab,ti | 25,192 | 2024.11.17 |  | #1 | Search TITLE-ABS-KEY ( coffee ) | 58,940 | 2024.11.17 |
| #2 | Search tea[Title/Abstract] | 42,935 | 2024.11.17 |  | #2 | Search 'tea':ab,ti | 51,874 | 2024.11.17 |  | #2 | Search TITLE-ABS-KEY (tea) | 101488 | 2024.11.17 |
| #3 | Search diet[Title/Abstract] | 436,354 | 2024.11.17 |  | #3 | Search 'diet':ab,ti | 587,607 | 2024.11.17 |  | #3 | Search TITLE-ABS-KEY (diet) | 1058,577 | 2024.11.17 |
| #4 | Search beverages[Title/Abstract] | 27,066 | 2024.11.17 |  | #4 | Search 'beverages':ab,ti | 32,004 | 2024.11.17 |  | #4 | Search TITLE-ABS-KEY (beverages) | 104,793 | 2024.11.17 |
| #5 | Search drinking[Title/Abstract] | 141,272 | 2024.11.17 |  | #5 | Search 'drinking':ab,ti | 190,171 | 2024.11.17 |  | #5 | Search TITLE-ABS-KEY (drinking) | 329,260 | 2024.11.17 |
| #6 | Search #1 OR #2 OR # 3 OR #4 OR #5 | 639,032 | 2024.11.17 |  | #6 | Search #1 OR #2 OR # 3 OR #4 OR #5 | 848,493 | 2024.11.17 |  | #6 | Search #1 OR #2 OR # 3 OR #4 OR #5 | 1,564,793 | 2024.11.17 |
| #7 | Search glioma[Title/Abstract] | 63,728 | 2024.11.17 |  | #7 | Search glioma:ab,ti | 85,446 | 2024.11.17 |  | #7 | Search TITLE-ABS-KEY (glioma) | 114,310 | 2024.11.17 |
| #8 | Search brain tumors[Title/Abstract] | 30,876 | 2024.11.17 |  | #8 | Search 'brain tumors':ab,ti | 43,854 | 2024.11.17 |  | #8 | Search TITLE-ABS-KEY ("brain tumors") | 153,892 | 2024.11.17 |
| #9 | Search brain cancer[Title/Abstract] | 5,425 | 2024.11.17 |  | #9 | Search 'brain cancer':ab,ti | 7,339 | 2024.11.17 |  | #9 | Search TITLE-ABS-KEY ("brain cancer") | 17,261 | 2024.11.17 |
| #10 | Search brain neoplasms[Title/Abstract] | 6,072 | 2024.11.17 |  | #10 | Search 'brain neoplasms':ab,ti | 939 | 2024.11.17 |  | #10 | Search TITLE-ABS-KEY ("brain neoplasms") | 120,491 | 2024.11.17 |
| #11 | Search cerebral cancer[Title/Abstract] | 24 | 2024.11.17 |  | #11 | Search 'cerebral cancer':ab,ti | 39 | 2024.11.17 |  | #11 | Search TITLE-ABS-KEY ("cerebral cancer") | 38 | 2024.11.17 |
| #12 | Search cerebral tumors[Title/Abstract] | 968 | 2024.11.17 |  | #12 | Search 'cerebral tumors':ab,ti | 1,600 | 2024.11.17 |  | #12 | Search TITLE-ABS-KEY ("cerebral tumors") | 2,701 | 2024.11.17 |
| #13 | Search cerebral neoplasms[Title/Abstract] | 207 | 2024.11.17 |  | #13 | Search 'cerebral neoplasms':ab,ti | 304 | 2024.11.17 |  | #13 | Search TITLE-ABS-KEY ("cerebral neoplasms") | 420 | 2024.11.17 |
| #14 | Search intracranial tumors[Title/Abstract] | 3,954 | 2024.11.17 |  | #14 | Search 'intracranial tumors':ab,ti | 5,575 | 2024.11.17 |  | #14 | Search TITLE-ABS-KEY ("intracranial tumors") | 11,517 | 2024.11.17 |
| #15 | Search intracranial cancer[Title/Abstract] | 20 | 2024.11.17 |  | #15 | Search 'intracranial cancer':ab,ti | 34 | 2024.11.17 |  | #15 | Search TITLE-ABS-KEY ("intracranial cancer") | 38 | 2024.11.17 |
| #16 | Search intracranial neoplasms[Title/Abstract] | 1,016 | 2024.11.17 |  | #16 | Search 'intracranial neoplasms':ab,ti | 1,469 | 2024.11.17 |  | #16 | Search TITLE-ABS-KEY ("intracranial neoplasms") | 1,690 | 2024.11.17 |
| #17 | Search central nervous system neoplasms[Title/Abstract] | 559 | 2024.11.17 |  | #17 | Search 'central nervous system neoplasms':ab,ti | 450 | 2024.11.17 |  | #17 | Search TITLE-ABS-KEY ("central nervous system neoplasms") | 8,206 | 2024.11.17 |
| #18 | Search central nervous system tumors[Title/Abstract] | 2,737 | 2024.11.17 |  | #18 | Search 'central nervous system tumors':ab,ti | 3,632 | 2024.11.17 |  | #18 | Search TITLE-ABS-KEY ("central nervous system tumors") | 15,064 | 2024.11.17 |
| #19 | Search central nervous system cancer[Title/Abstract] | 210 | 2024.11.17 |  | #19 | Search 'central nervous system cancer':ab,ti | 266 | 2024.11.17 |  | #19 | Search TITLE-ABS-KEY ("central nervous system cancer") | 2,014 | 2024.11.17 |
| #20 | Search #7 OR #8 OR # 9 OR #10 OR #11 OR #12 OR #13 OR #14 OR #15 OR #16 OR #17 OR #18 OR #19 | 102,278 | 2024.11.17 |  | #20 | Search #7 OR #8 OR # 9 OR #10 OR #11 OR #12 OR #13 OR #14 OR #15 OR #16 OR #17 OR #18 OR #19 | 135,123 | 2024.11.17 |  | #20 | Search #7 OR #8 OR # 9 OR #10 OR #11 OR #12 OR #13 OR #14 OR #15 OR #16 OR #17 OR #18 OR #19 | 270,579 | 2024.11.17 |
| #21 | Search #6 AND #20 | 458 | 2024.11.17 |  | #21 | Search #6 AND #20 | 657 | 2024.11.17 |  | #21 | Search #6 AND #20 | 1,687 | 2024.11.17 |

**Supplementary Table 2 Basic characteristic.**

| Study | Country | Cohort name | Exposure | Exposure category | RR (95% CI) | Adjustment for potentially confounding factors |
| --- | --- | --- | --- | --- | --- | --- |
| Efird et al. 2004 [20] | United States | KPMCP-NC | Coffee | < 1 cups/day | 1.0 (Ref.) | Age, cigarettes, cigars, pipes, sex, race, education, alcohol. |
|  |  |  |  | 1-3 cups/day | 1.1 (0.7-1.8) |  |
|  |  |  |  | 4-6 cups/day | 1.6 (0.9-2.8) |  |
|  |  |  |  | ≥7 cups/day | 1.7 (0.8-3.6) |  |
| Michaud et al. 2010 [21] | European | EPIC | Coffee | Q1 | 1.0 (Ref.) | Age, country, sex, smoking status, BMI, and education |
|  |  |  |  | Q2 | 1.21 (0.86-1.71) |  |
|  |  |  |  | Q3 | 1.15 (0.81-1.63) |  |
|  |  |  |  | Q4 | 1.27 (0.87-1.84) |  |
|  |  |  |  | Q5 | 0.98 (0.67-1.41) |  |
|  |  |  | Tea | Q1 | 1.0 (Ref.) | Age, country, sex, smoking status, BMI, and education |
|  |  |  |  | Q2 | 0.76 (0.52-1.10) |  |
|  |  |  |  | Q3 | 0.80 (0.55-1.17) |  |
|  |  |  |  | Q4 | 1.05 (0.75-1.48) |  |
| Dubrow et al. 2012 [24] | United States | NIH-AARP | Coffee | 0 cup/day | 1.0 (Ref.) | Age, sex, race/ethnicity, energy intake, height, fruit and vegetable intake, nitrite intake from plant sources. |
|  |  |  |  | >0 to <1 cup/day | 0.82(0.62-1.07) |  |
|  |  |  |  | 1 cup/day | 0.96(0.74-1.24） |  |
|  |  |  |  | 2 to 3 cups/day | 1.04(0.83-1.30) |  |
|  |  |  |  | 4 to 5 cups/day | 0.77(0.57-1.03) |  |
|  |  |  |  | ≥6 cups/day | 1.04(0.70-1.55) |  |
|  |  |  | Tea | 0 cup/day | 1.0 (Ref.) | Age, sex, race/ethnicity, energy intake, height, fruit and vegetable intake, and nitrite intake from plant sources |
|  |  |  |  | >0 to ≤0.5 cups/day | 0.81(0.65-1.02) |  |
|  |  |  |  | >0.5 to ≤1 cups/day | 0.88(0.69-1.12) |  |
|  |  |  |  | >1 to ≤2 cups/day | 0.80(0.60-1.06) |  |
|  |  |  |  | >2 to ≤3 cups/day | 0.96(0.74-1.23) |  |
|  |  |  |  | >3 cups/day | 0.75(0.57-1.00) |  |
| Nelson et al. 2012 [25] | United States | HHP/HAAS | Coffee | 0 cup/day | 1.0 (Ref.) | Age, education, triceps, and skinfold thickness |
|  |  |  |  | 1-3 oz/day | 1.83 (0.21–16.25) |  |
|  |  |  |  | ≥ 4 oz/day | 0.89 (0.08-10.02) |  |
|  |  |  | Tea | 0 cup/day | 1.0 (Ref.) | Age, education, triceps, and skinfold thickness |
|  |  |  |  | 1-3 oz/day | 0.79 (0.14-4.47) |  |
|  |  |  |  | ≥ 4 oz/day | 1.21 (0.22–6.76) |  |
| Hashibe et al. 2015 [26] | United States | PLCO | Coffee | < 1 cup/day | 1.0 (Ref.) | Age, sex, race, education, cigarette pack-years, alcohol drinking frequency. |
|  |  |  |  | 1-1.9 cups/day | 0.86 (0.48–1.55) |  |
|  |  |  |  | ≥ 2 cups/day | 0.76 (0.50–1.17) |  |
|  |  |  | Tea | < 1 cup/day | 1.0 (Ref.) | Age, sex, race, education, cigarette pack-years, alcohol drinking frequency. |
|  |  |  |  | ≥ 1 cups/day | 1.04 (0.65–1.66) |  |
| Ogawa et al. 2016 [27] | Japan | JPHCS | Coffee | < 4 cups/week | 1.0 (Ref.) | Age, sex, BMI, pack years of cigarettes, alcohol intake, green tea, and past history of allergy and diabetes mellitus. |
|  |  |  |  | 1-2 cups/day | 1.05 (0.57–1.92) |  |
|  |  |  |  | ≥3 cups/day | 0.55 (0.17-1.84) |  |
|  |  |  | Tea | < 4 cups/week | 1.0 (Ref.) |  |
|  |  |  |  | 1-2 cups/day | 1.12 (0.51–2.43) |  |
|  |  |  |  | ≥3 cups/day | 1.05 (0.54–2.05) |  |
| Kuan et al. 2019 [28] | The UK | MWS | Coffee | Lowest fourth | 1.0 (Ref.) | Age or birth year, sex, race, marital status, height, BMI, smoking, alcohol intake, education, physical activity, region of residence, social deprivation; and additionally, parity, OC, and MH use |
|  | United States | NIH-AARP |  | Second fourth | 1.02 (0.90–1.15) | for women |
|  | United States | PLCO |  | Third fourth | 1.03 (0.91–1.17) |  |
|  |  |  |  | Highest fourth | 1.01 (0.89–1.16) |  |
|  |  |  | Tea | Lowest fourth | 1.0 (Ref.) | Age or birth year, sex, race, marital status, height, BMI, smoking, alcohol intake, education, physical activity, region of residence, social deprivation; and additionally, parity, OC, and MH use |
|  |  |  |  | Second fourth | 1.00 (0.89–1.13) | for women |
|  |  |  |  | Highest fourth | 0.97 (0.86–1.09) |  |
|  |  |  |  | Highest fourth | 1.01 (0.89–1.14) |  |
| Creed et al 2020 [29] | The UK | The UK Biobank | Coffee | 0 cup/day | 1.0 (Ref.) | Age, gender, race, education, smoking status, weekly alcohol consumption, body mass index, and tea. |
|  |  |  |  | 0-2 cups/day | 0.89 (0.69-1.15) |  |
|  |  |  |  | 2-4 cups/day | 0.94 (0.70-1.27) |  |
|  |  |  |  | 4 cups/day | 0.71 (0.48-1.05) |  |
|  |  |  | Tea | 0 cup/day | 1.0 (Ref.) | Age, gender, race, education, smoking status, weekly alcohol consumption, body mass index, and coffee. |
|  |  |  |  | 0-2 cups/day | 0.72 (0.53-0.98) |  |
|  |  |  |  | 2-4 cups/day | 0.76 (0.57-1.02) |  |
|  |  |  |  | 4 cups/day | 0.68 (0.50-0.93) |  |
| Cote et al. 2020 [30] | United States | NHS | Coffee | <1 cup /week | 1.0 (Ref.) | Age, total caloric intake, BMI, tea, and smoking status. |
|  |  |  |  | ≥1 cup/week to ≤1.5 cups/day | 0.86 (0.55–1.35) |  |
|  |  |  |  | >1.5 to ≤2.5 cups/day | 1.12 (0.73.–1.73) |  |
|  |  |  |  | >2.5 to ≤4 cups/day | 1.37 (0.88–2.13) |  |
|  |  |  |  | >4 cups/day | 1.19 (0.71–1.99) |  |
|  |  |  | Tea | <1 cups /week | 1.0 (Ref.) | Age, total caloric intake, BMI, coffee, and smoking status. |
|  |  |  |  | ≥1/week to≤1.5 cups/day | 0.92 (0.70–1.21) |  |
|  |  |  |  | >1.5 to ≤2 cups/day | 0.74 (0.47–1.16) |  |
|  |  |  |  | >2 cups/day | 0.82 (0.48–1.40) |  |
| Cote et al. 2020 [30] | United States | NHSII | Coffee | <1 cup /week | 1.0 (Ref.) | Age, total caloric intake, BMI, tea, and smoking status. |
|  |  |  |  | ≥1 cups/week to ≤1.5 cups/day | 0.80 (0.45-1.43) |  |
|  |  |  |  | >1.5 to ≤2.5 cups/day | 0.92 (0.51-1.66) |  |
|  |  |  |  | >2.5 to ≤4 cups/day | 0.67 (0.33-1.35) |  |
|  |  |  |  | >4 cups/day | 0.70 (0.26-1.88) |  |
|  |  |  | Tea | <1 cup/week | 1.0 (Ref.) | Age, total caloric intake, BMI, coffee, and smoking status. |
|  |  |  |  | ≥1/week to≤1.5 cups/day | 0.74 (0.46-1.19) |  |
|  |  |  |  | >1.5 to ≤2 cups/day | 0.97 (0.48-1.95) |  |
|  |  |  |  | >2 cups/day | 0.66 (0.26-1.69) |  |
| Cote et al. 2020 [30] | United States | HPFS | Coffee | <1 cup/week | 1.0 (Ref.) | Age, total caloric intake, BMI, tea, and smoking status. |
|  |  |  |  | ≥1 cups/week to ≤1.5 cups/day | 0.80 (0.53-1.21) |  |
|  |  |  |  | >1.5 to ≤2.5 cups/day | 0.90 (0.58-1.38) |  |
|  |  |  |  | >2.5 to ≤4 cups/day | 0.99 (0.63-1.54) |  |
|  |  |  |  | >4 cups/day | 0.70 (0.38-1.30) |  |
|  |  |  | Tea | <1 cup/week | 1.0 (Ref.) | Age, total caloric intake, BMI, coffee, and smoking status. |
|  |  |  |  | ≥1/week to≤1.5 cups/day | 0.97 (0.72-1.30) |  |
|  |  |  |  | >1.5 to ≤2 cups/day | 0.82 (0.45-1.49) |  |
|  |  |  |  | >2 cups/day | 0.68 (0.29-1.58) |  |

Abbreviation: KPMCP-NC, Kaiser Permanente Medical Care Program of Northern California; EPIC (Denmark, Italy, the Netherlands, Norway, Spain, Sweden, and the United Kingdom. For France, Germany, and Greece), the European Prospective Investigation into Cancer and Nutrition; NIH-AARP, the NIH-AARP Diet and Health Study; HHP/HAAS, the Honolulu Heart Program/ the Honolulu- Asia Aging Study; PLCO, the Prostate, Lung, Colorectal, and Ovarian cancer screening trial; JPHCS, the Japan Public Health Center-Based Prospective Study; HPFS, Health Professionals Follow-up Study; NHS, Nurses’ Health Study; NHSII, Nurses’ Health Study II; MWS, Million Women Study; NDI, national death index; BMI, body mass index; OC, oral contraceptive; MH, use of menopausal hormone; NR, not report.

**Supplementary Table 3 Sensitivity analyses results for coffee.**

| Study omitted | HR (95% CI) and *P* | I^2^ | *P* _heterogeneity_ |
| --- | --- | --- | --- |
| Efird et al. 2004 [20] | 0.96(0.86-1.08) 0.521 | 0.0% | 0.567 |
| Michaud et al. 2010 [21] | 0.98(0.87-1.10) 0.677 | 11.5% | 0.341 |
| Nelson et al. 2012 [25] | 0.98(0.87-1.09) 0.670 | 11.4% | 0.341 |
| Ogawa et al. 2016 [27] | 0.98(0.88-1.10) 0.734 | 0.1% | 0.428 |
| Kuan et al. 2019 [28] | 0.90(0.73-1.10) 0.308 | 0.5% | 0.425 |
| Creed et al. 2020 [29] | 1.00(0.89-1.13) 0.952 | 0.0% | 0.642 |
| Cote et al. 2020 NHS [30] | 0.97(0.86-1.08) 0.558 | 4.3% | 0.397 |
| Cote et al. 2020 NHSII [30] | 0.98(0.88-1.10) 0.725 | 6.3% | 0.382 |
| Cote et al. 2020 HPFS [30] | 0.99(0.88-1.11) 0.820 | 0.0% | 0.445 |

**Supplementary Table 4 Sensitivity analyses results for tea.**

| Study omitted | HR (95% CI) and *P* | I^2^ | *P* _heterogeneity_ |
| --- | --- | --- | --- |
| Michaud et al. 2010 [21] | 0.94(0.85-1.05) 0.297 | 14.5% | 0.319 |
| Nelson et al. 2012 [25] | 0.95(0.86-1.06) 0.356 | 17.7% | 0.295 |
| Ogawa et al. 2016 [27] | 0.95(0.86-1.06) 0.347 | 17.6% | 0.296 |
| Kuan et al. 2019 [28] | 0.83(0.69-1.01) 0.057 | 0.0% | 0.605 |
| Creed et al. 2020 [29] | 0.99(0.89-1.11) 0.921 | 0.0% | 0.896 |
| Cote et al. 2020 NHS [30] | 0.96(0.86-1.07) 0.435 | 14.9% | 0.316 |
| Cote et al. 2020 NHSII [30] | 0.96(0.86-1.06) 0.414 | 11.3% | 0.343 |
| Cote et al. 2020 HPFS [30] | 0.96(0.86-1.06) 0.421 | 11.0% | 0.345 |

**Supplementary Table 5 The quality of included studies based on Newcastle-Ottawa Scale.**

|  |  |  | Selection |  |  |  | Comparability |  | Outcome |  |  |
| --- | --- | --- | --- | --- | --- | --- | --- | --- | --- | --- | --- |
| Reference | Cohort name | Representativeness of the exposed cohort | Selection of the non exposed cohort | Ascertainment of exposure | Demonstration that outcome of interest was not present at start of study |  | Comparability of cohorts on the basis of the design or analysis |  | Assessment of outcome | Was follow-up long enough for outcomes to occur | Adequacy of follow up of cohorts |
| Efird et al. 2004 [20] | KPMCP-NC | * | * | ? | * |  | * |  | * | * | * |
| Michaud et al. 2010 [21] | EPIC | * | * | * | * |  | * |  | * | * | * |
| Dubrow et al. 2012 [24] | NIH-AARP | * | * | * | * |  | * |  | * | * | * |
| Nelson et al. 2012 [25] | HHP/HAAS | * | * | * | * |  | * |  | * | * | * |
| Hashibe et al. 2015 [26] | PLCO | * | * | * | * |  | * |  | * | * | * |
| Ogawa et al. 2016 [27] | JPHCS | * | * | * | * |  | * |  | * | * | * |
| Kuan et al. 2019 [28] | MWS | * | * | * | * |  | * |  | * | * | * |
|  | NIH-AARP | * | * | * | * |  | * |  | * | * | * |
|  | PLCO | * | * | * | * |  | * |  | * | * | * |
| Creed et al 2020 [29] | The UK Biobank | * | * | ? | * |  | * |  | * | * | * |
| Cote et al. 2020 [30] | NHS | * | * | * | * |  | * |  | * | * | * |
|  | NHSII | * | * | * | * |  | * |  | * | * | * |
|  | HPFS | * | * | * | * |  | * |  | * | * | * |

Abbreviation: KPMCP-NC, Kaiser Permanente Medical Care Program of Northern California; EPIC (Denmark, Italy, the Netherlands, Norway, Spain, Sweden, and the United Kingdom. For France, Germany, and Greece), the European Prospective Investigation into Cancer and Nutrition; NIH-AARP, the NIH-AARP Diet and Health Study; HHP/HAAS, the Honolulu Heart Program/ the Honolulu- Asia Aging Study; PLCO, the Prostate, Lung, Colorectal, and Ovarian cancer screening trial; JPHCS, the Japan Public Health Center-Based Prospective Study; HPFS, Health Professionals Follow-up Study; NHS, Nurses’ Health Study; NHSII, Nurses’ Health Study II; MWS, Million Women Study.

**Supplementary Table 6 The quality of meta-evidence based on Grade Scoring System.**

| Exposure |  | Quality assessment | | | | | | |  | Quality |
| --- | --- | --- | --- | --- | --- | --- | --- | --- | --- | --- |
|  |  | Design | Risk of bias | Inconsistency | Indirectness | Imprecision | Publication bias | Increase grade consideration |  |  |
| Coffee intake |  | Cohort study | Not serious | Not serious (I^2^=0.0%) | Not serious | Serious* | Undetected | **/** |  | Very Low |
| Tea intake |  | Cohort study | Not serious | Not serious (I^2^=0.49%) | Not serious | Serious* | Undetected | Dose response gradient |  | Low |

* This meta-analysis was based on small number of glioma cases.

**Supplementary Table 7 Comparison with Previous Meta-analyses**

| Characteristic | Malerba et al. [31] | Zhang et al. [32] | Song et al. [34] | Zhao et al. [35] | Pranata et al. [36] | Zhang et al. [37] | The present study |
| --- | --- | --- | --- | --- | --- | --- | --- |
| Search date updated | September 2012 | October 2013 | November 1, 2018, | June 2020 | October 1, 2020 | October 11, 2021 | September 24, 2024 |
| Retrospective studies included (Reference number) | Yes (15, 16) |  | Yes (15,19) |  | Yes (15,16,19) | Yes (15,16,19) |  |
| Prospective studies included (Reference number) | Yes (20,21,22,24) | Yes (21,22,24) | Yes (20-27) | Yes (21,24-26,29,30) | Yes (20,21,23-27,29,30) | Yes (20-27,29,30) | Yes (20,21,24-30) |
| Glioma cases | 2100 | 1582 | 2336 | 2383 | 2987 | 3433 | 3896 |
| Main finding for coffee (highest vs. lowest) | 1.01 (95 % CI 0.83–1.22) |  | 0.76 (95% CI 0.55–0.97) |  | 0.77(95% CI 0.55-1.03) | 0.81 (95% CI 0.62–1.06) | 0.97 (95% CI 0.87-1.09) |
| Dose-response analysis for coffee | Linear:  Increment of one cup/day  1.0 (95% CI: 0.96-1.05) |  |  |  | Borderline non-linearity  Increment of one cup/day  0.97(95% CI 0.94-0.99) |  | Linearity |
| Main finding for tea (highest vs. lowest) | 0.88 (95 % CI 0.69–1.12) | 0.83 (95 % CI 0.68−1.02) | 0.85 (95% CI 0.68–1.05) | 0.81(95% CI: 0.70-0.95) | 0.84 (95% CI 0.71-0.98) | 0.82 (95% CI: 0.71–0.93) | 0.95 (95% CI 0.86-1.06) |
| Dose-response analysis for tea |  | Linear**?**  Increment of one cup/day  0.98 (95 % CI 0.95−1.01) |  | Linear**?**  Increment of one cup/day  0.98(95% CI 0.94-.02) | Linear:  Increment of one cup/day  0.97(95% CI 0.94-1.00) | Linear | Non-linearity: The pronounced inverse association observed for individuals consuming more than 2.5 cups of tea per day. No evident reduction in glioma risk was observed with further increases in tea consumption beyond this level. |


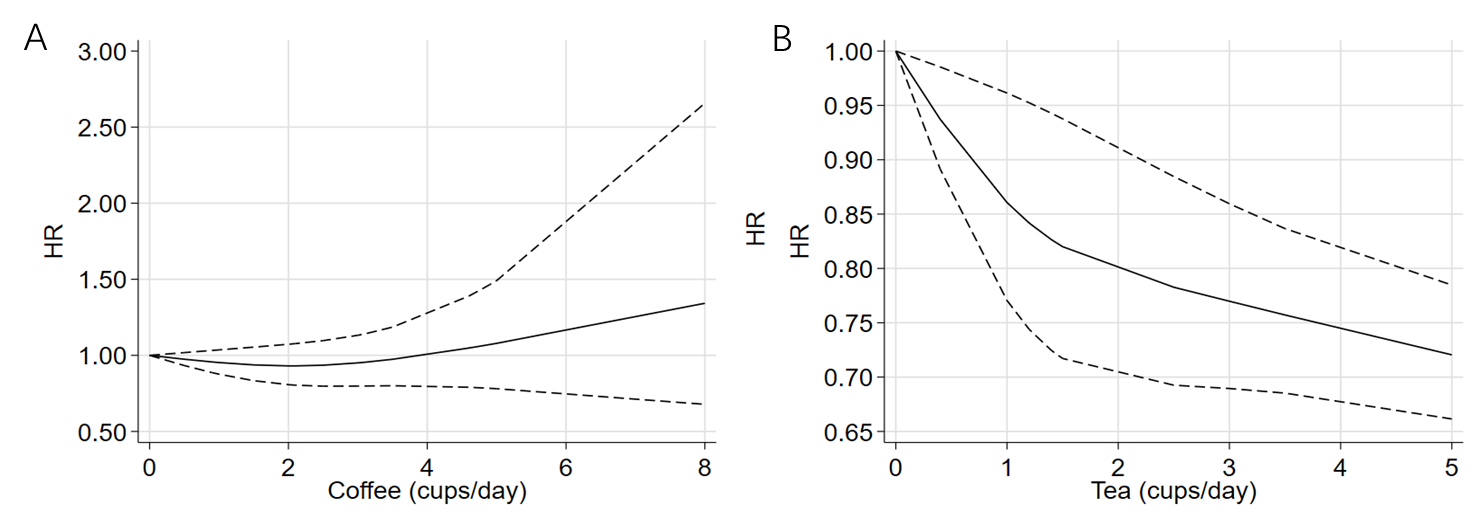


**Supplementary Figure S1** Dose-response relationship limited to those studies adjusted for smoking. A. coffee; B, tea.
